# Polyantigenic Interferon-γ Responses Are Associated with Protection from TB among HIV-Infected Adults with Childhood BCG Immunization

**DOI:** 10.1371/journal.pone.0022074

**Published:** 2011-07-20

**Authors:** Timothy Lahey, Brian K. Mitchell, Robert D. Arbeit, Siddharth Sheth, Mecky Matee, C. Robert Horsburgh, Todd MacKenzie, Lillian Mtei, Muhammad Bakari, Jenni M. Vuola, Kisali Pallangyo, C. Fordham von Reyn

**Affiliations:** 1 Dartmouth Medical School, Lebanon, New Hampshire, United States of America; 2 The Dartmouth Institute for Health Policy and Clinical Practice, Lebanon, New Hampshire, United States of America; 3 Tufts University School of Medicine, Boston, Massachusetts, United States of America; 4 Muhimbili University of Health and Allied Sciences, Dar es Salaam, Tanzania; 5 Boston University School of Public Health, Boston, Massachusetts, United States of America; 6 National Public Health Institute, Helsinki, Finland; University of Cape Town, South Africa

## Abstract

**Background:**

Surrogate immunologic markers for natural and vaccine-mediated protection against tuberculosis (TB) have not been identified.

**Methods:**

HIV-infected adults with childhood BCG immunization entering the placebo arm of the DarDar TB vaccine trial in Dar es Salaam, Tanzania, were assessed for interferon gamma (IFN-γ) responses to three mycobacterial antigen preparations – secreted *Mycobacterium tuberculosis* antigens 85 (Ag85), early secretory antigenic target 6 (ESAT-6) and polyantigenic whole cell lysate (WCL). We investigated the association between the number of detectable IFN-γ responses at baseline and the subsequent risk of HIV-associated TB.

**Results:**

During a median follow-up of 3.3 years, 92 (9.4%) of 979 placebo recipients developed TB. The incidence of TB was 14% in subjects with no detectable baseline IFN-γ responses vs. 8% in subjects with response to polyantigenic WCL (P = 0.028). Concomitant responses to secreted antigens were associated with further reduction in the incidence of HIV-associated TB. Overall the percentage of subjects with 0, 1, 2 and 3 baseline IFN-γ responses to mycobacterial preparations who developed HIV-associated TB was 14%, 8%, 7% and 4%, respectively (P = 0.004). In a multivariate Cox regression model, the hazard of developing HIV-associated TB was 46% lower with each increment in the number of detectable baseline IFN-γ responses (P<0.001).

**Conclusions:**

Among HIV-infected adults who received BCG in childhood and live in a TB-endemic country, polyantigenic IFN-γ responses are associated with decreased risk of subsequent HIV-associated TB.

**Trial Registration:**

ClinicalTrials.gov NCT0052195

## Introduction

The existing TB vaccine, bacille Calmette Guérin (BCG), provides protection against tuberculosis (TB) when given to mycobacteria-naïve newborns, but protection is incomplete and wanes in adulthood [1], [2], [3], [4], [5], [6], [7], [8], [9], [10]. Thus, the development of new and more effective primary and booster vaccines against TB is a critical global health priority [11].

TB vaccine candidates now being tested for safety and efficacy in humans include pauci-antigenic subunit vaccines encoding one or two immunodominant mycobacterial antigens, and polyantigenic whole cell mycobacterial vaccines such as recombinant BCG and inactivated *Mycobacterium vaccae* [12], [13]. Animal models suggest that vaccines encoding one or two antigens provide protection equivalent to BCG [14], [15]. However, in epidemiologic studies and TB vaccine trials in humans, protection against TB has only been observed either after natural infection with *M. tuberculosis* or non-tuberculous mycobacteria (NTM) [16], [17], [18], [19], or after immunization with live or inactivated whole cell mycobacterial vaccines [10], [20], [21], [22], [23], [24], [25], [26], [27]. All of these situations involve polyantigenic challenge with diverse mycobacterial components.

Previously we have shown that HIV-infected subjects with childhood BCG immunization living in a TB-endemic country and followed prospectively have a reduced risk of developing TB if they exhibit detectable baseline interferon gamma (IFN-γ) responses to mycobacterial antigens [28]. In the present study, using the immunological database developed in our previous study, we explored whether the number of baseline IFN-γ responses targeting mycobacterial antigens was associated with protection against TB in BCG-immunized adults with HIV infection.

## Methods

### Human research conduct

We followed human experimentation guidelines of the United States Department of Health and Human Services in the research protocol for this study, which was approved by the Committee for the Protection of Human Subjects at Dartmouth College and the Research Ethics Committee of the Muhimbili University of Health and Allied Sciences. Subjects provided written informed consent in English or Kiswahili with the help of a translator as needed. This study was registered through the National Institutes of Health (NCT00052195).

### Study subjects

The DarDar trial was a phase III randomized placebo-controlled double-blind trial of a novel booster vaccine for the prevention of HIV-associated TB among adults in Dar es Salaam, Tanzania [13]. Enrollment occurred from 2001 to 2005, and study follow up continued through January 2008. Subjects were eligible for enrollment if they had two positive enzyme linked immunosorbent assay (ELISA) tests for HIV, a CD4 count ≥200/mm3, and a BCG scar. At baseline, all subjects were evaluated with history, physical examination, single view chest x-ray, sputum acid fast bacillus (AFB) smear, sputum mycobacterial culture, and blood mycobacterial culture. Subjects with active TB were excluded from enrollment. Subjects were randomized to receive five intradermal doses of either heat inactivated *Mycobacterium vaccae* or matched saline placebo. Subjects who received vaccine exhibited protection against definite tuberculosis [13]. For the present study, we confined our analyses to subjects who received placebo.

### Clinical surveillance for TB disease

After randomization, we evaluated subjects for active TB disease by interim history and physical examination at months 2, 4, and 6, and every three months thereafter. In addition, at any time subjects presented with two or more weeks of fever, cough or weight loss, they underwent evaluation for active TB via a single view chest x-ray, three sputum collections for AFB smear and mycobacterial culture, phlebotomy for mycobacterial blood culture, and any additional studies as clinically indicated (e.g., cultures of other body fluids or tissue biopsies).

### Definitions of TB

A three-person blinded adjudication panel reviewed all episodes of illness evaluated for active TB and designated subjects as having definite or probable TB according to rigorous published study definitions [13].

### Assays of mycobacterial immune responses

All subjects underwent in vivo and in vitro assessments of immune responses to mycobacteria prior to vaccination. These assessments included mycobacterial skin tests, an assay of interferon gamma (IFN-γ) release, and a standard tritiated thymidine lymphocyte proliferation assay (LPA).

### Skin tests

Tuberculin skin tests (TST) were performed on all subjects by intradermal injection of purified protein derivative, PPD (0.1 mL, RT-23, State Serum Institute, Copenhagen) on the forearm; trained personnel measured the size of skin induration at the site after 48–72 hours. We considered reactions of ≥5 mm positive, and offered isoniazid treatment to all such subjects [29].

### IFN-γ release assay

Freshly isolated and ficolled peripheral blood mononuclear cells (PBMC) were incubated with study antigens for five days in IFN-γ assays. After five days, centrifuged cell supernatants were frozen and sent to the United States for later IFN-γ level measurement using a standard IFN-γ enzyme linked immunosorbent assay (ELISA; R&D Systems, Minneapolis, MN). Study antigens were medium alone (negative control), 1 mcg/ml *M. tuberculosis* early secretory antigenic target 6 (ESAT-6), 0.5 mcg/ml *M. tuberculosis* antigen 85 (Ag85), or 0.5 mcg/ml *M. tuberculosis* whole cell lysate (WCL), with all antigens acquired through NIH, NIAID Contract No. HHSN266200400091C “Tuberculosis Vaccine Testing and Research Materials” awarded to Colorado State University. IFN- γ assays were considered valid if the IFN-γ level for the positive control antigen, PHA (Sigma, St. Louis, MO; 2.5 µg/ml), was greater than 300 pg/mL. IFN-γ responses to mycobacterial antigens were considered positive if the IFN- γ level was greater than or equal to two standard deviations above the mean of the negative control condition.

#### LPA

LPA assays were conducted on the same PBMC used in the IFN-γ assay using a standard five-day ^3^H-thymidine incorporation method. After incubation with study antigens, 20 µl of 50 µCi/ml 3H-thymidine was added to wells for 24 hours, and then cells were harvested onto filter paper and sent to the National Public Health Institute in Helsinki, Finland, for data acquisition on a scintillation counter. Results were expressed as a proliferation index (PI; defined as counts per minute [CPM] of antigen stimulated cells divided by CPM of unstimulated cells). LPA assays were considered valid if the PI for the positive control antigen phytohemagglutinin (PHA; 2.5 µg/ml) was ≥3. LPA responses to mycobacterial antigens were considered positive if the PI was greater than or equal to five.

### Statistical analysis

We categorized subjects according to the number of antigens against which baseline IFN-γ responses, or LPA responses, were targeted. Then, we compared demographic data and TB incidence according to the number of antigens targeted by baseline immune responses using a two-tailed Mann-Whitney *U* test, student's t test or chi squared test as appropriate. A Kruskal-Wallis test was used specifically to compare demographic factors between subjects according to the number of mycobacterial antigens targeted by baseline immune responses. We then constructed a multivariate Cox proportional hazards regression model of the hazard of TB during prospective follow up, adjusting for age, baseline CD4 count, previous TB treatment, and baseline TST status. We confirmed that the proportional hazards assumption was not violated using log-log plots and Schoenfeld residuals. For statistical analyses, we used STATA 9 (College Station, TX).

## Results

### Subject characteristics

**Figure 1:**
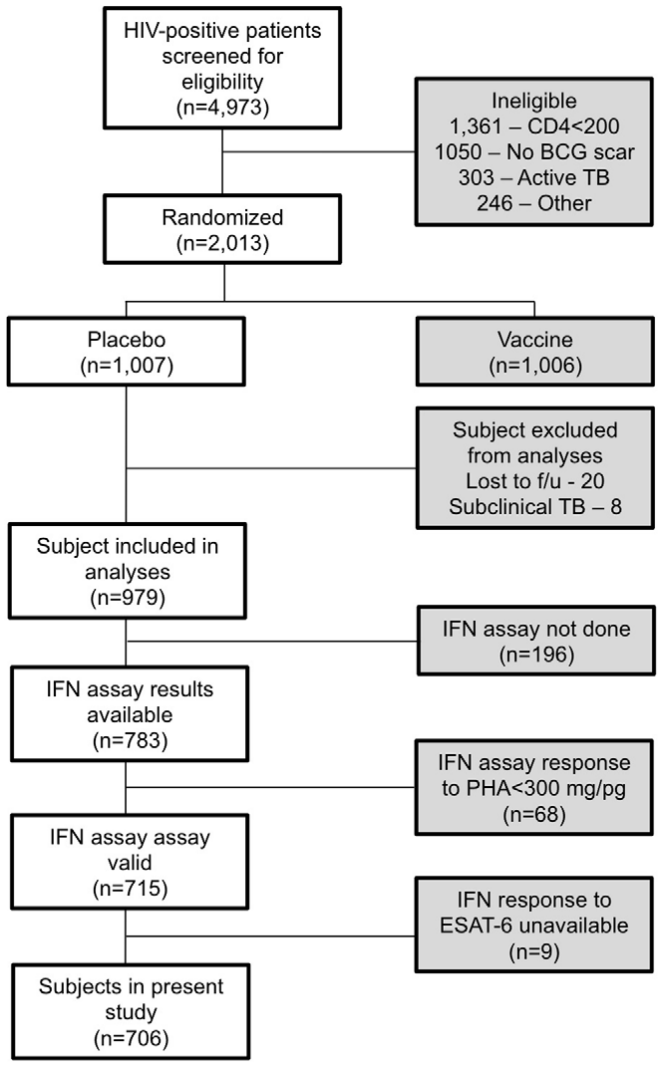
Study CONSORT diagram. ESAT-6, early secreted antigenic target 6; f/u, follow up; IFN, interferon gamma; PHA, phytohemagglutinin positive control; TB, tuberculosis.

Among the 979 HIV-infected placebo subjects in this study, all had a BCG scar and a baseline CD4 count ≥200/mm^3^ at study entry. We diagnosed 92 causes of definite and probable TB during a median follow-up of 3.3 years. As we showed previously [28], subjects who developed HIV-associated TB had lower CD4 counts, had higher HIV viral loads, were more likely to have a positive TST, and were more likely to have been treated for TB previously. Figure 1 depicts the study CONSORT diagram.

### Relation of multiple IFN-γ responses to TB risk in univariate analyses

**Figure 2:**
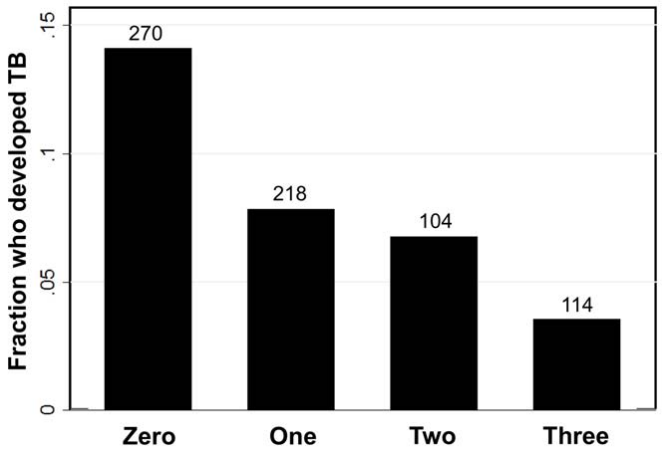
Fraction of subjects who developed HIV-associated tuberculosis (TB) according to the number of baseline interferon gamma (IFN-γ) responses detected against mycobacterial preparations.

Table 1 presents percentage of subjects who developed HIV-associated TB during follow up according to which mycobacterial antigen preparations were targeted by baseline IFN-γ responses. Baseline IFN-γ responses were observed most commonly to polyantigenic WCL; only 2% of subjects had IFN-γ responses to one or both secreted antigens in the absence of a response to WCL. Among subjects with no IFN-γ responses at baseline, 14% developed incident TB. Response to WCL was associated with substantial reduction in the incidence of HIV-associated TB (8%), as we showed previously [28]. The incidence of TB decreased progressively among subjects with additional detectable IFN-γ responses, and was lowest (4%) among subjects with baseline IFN-γ responses to all three mycobacterial antigen preparations (Figure 2).

**Table 1:** Percentage of subjects who developed HIV-associated TB during longitudinal follow up according to antigen specificity of baseline IFN-γ responses.

Number of baseline IFN-γ responses	Antigen	n	Percent who developed TB
Zero	None	270	14%
One	ESAT-6	4	25%
	Ag85	6	0%
	WCL	208	8%
Two	ESAT-6 + Ag85	1	0%
	WCL + ESAT-6	66	5%
	WCL + Ag85	37	11%
Three	WCL + Ag85 + ESAT-6	114	4%
	Total	706	9%

Ag85, antigen 85; ESAT-6, early secreted antigenic target 6; IFN-γ, interferon gamma; TB, tuberculosis; WCL, whole cell lysate.

### Univariate predictors of multiple IFN-γ responses

Subjects with a greater number of baseline IFN-γ responses against mycobacterial antigens had higher baseline CD4 counts, lower HIV viral loads, and were more likely to have a have a positive TST (Table 2).

**Table 2:** Baseline subject characteristics according to number of detectable baseline IFN-γ responses to mycobacterial antigens.

Characteristic	Number of IFN-γ responses to mycobacterial antigens				P-value
	0	1	2	3	
Age, mean years (SD)	33.2 (8.0)	32.4 (7.8)	34.4 (8.0)	32.3 (7.4)	0.15
Male, % (N)	23.1 (73/316)	24.2 (56/231)	25.5 (28/110)	25.2 (29/115)	0.948
Prior treatment for TB, % (N)	9.2 (29/316)	9.1 (21/231)	7.3 (8/110)	10.4 (12/115)	0.874
On HIV treatment at baseline, % (N)	3.8 (12/316)	4.3 (10/231)	3.6 (4/110)	3.5 (4/115)	0.978
Positive TST, % (N)	11.3 (35/310)	39.3 (90/229)	48.6 (52/107)	71.7 (81/113)	<0.001
Baseline CD4 count, mean cells/µL (SD)	419 (203)	507 (230)	547 (272)	570 (277)	<0.001
Baseline HIV viral load, mean log_10_ (SD)	4.1 (0.8)	3.9 (0.8)	3.5 (1.1)	3.4 (0.9)	<0.001

P values are derived via Mann-Whitney *U* tests, student's t tests and chi squared tests as appropriate.

SD, standard deviation; TB, tuberculosis; TST, tuberculin skin test.

#### Relation of multiple IFN-γ responses to TB risk in multivariate analyses

**Figure 3:**
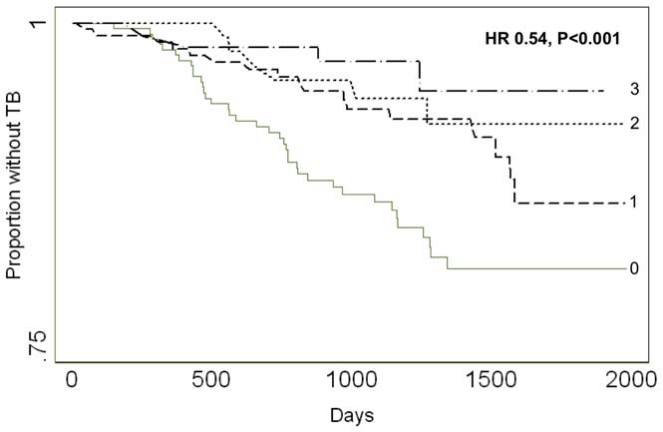
Survival without HIV-associated tuberculosis (TB) according to number of baseline interferon gamma (IFN-γ) responses detected against mycobacterial preparations. The hazard of HIV-associated TB fell 46% with each increment in the number of detectable baseline IFN-γ responses against mycobacterial preparations. HR, hazard ratio; TB, tuberculosis.

We used a multivariate Cox regression model to assess the hazard of developing HIV-associated TB relative to the number of detectable baseline IFN-γ responses against mycobacterial antigen preparations. In unadjusted analyses, the hazard decreased significantly with each incremental increase in the number of baseline IFN-γ responses (hazard ratio [HR] 0.62, 95% confidence intervals [CI] 0.47-0.81, P = 0.001). This relationship remained significant after adjusting for age, baseline CD4 count, previous TB treatment and a positive TST (HR 0.54, 95% CI 0.38–0.75, P<0.001; Figure 3). Compared to subjects with baseline IFN-γ responses to WCL alone, subjects responding to both WCL and ESAT-6 exhibited a trend toward enhanced protection from HIV-associated TB compared (HR 0.43, 95% CI 0.18−1.06, P = 0.069), whereas subjects responding to both WCL and Ag85 did not (HR 0.85, 95% CI 0.35−2.05, P = 0.715).

In supplemental analyses, the number of baseline IFN-γ responses to mycobacterial antigens remained significantly associated with the hazard of HIV-associated TB both among subjects with a negative baseline TST (HR 0.42, 95% CI 0.21-0.84, P = 0.014) and among subjects with a positive baseline TST (HR 0.58, 95% CI 0.39−0.87, P = 0.008). In the smaller subset of 234 subjects for whom baseline HIV viral load data are available, there was a trend toward greater protection from HIV-associated TB among subjects with a greater number of baseline IFN-γ responses when adjusting for baseline HIV viral load (HR 0.58, 95% CI 0.33−1.03, P = 0.063).

### No relation of number of baseline LPA responses to TB risk

Like subjects with a greater number of baseline IFN-γ responses to mycobacterial antigens, subjects with a greater number of baseline LPA responses to mycobacterial antigens had higher baseline CD4 counts (P = 0.026), lower HIV viral loads (P = 0.010), and were more likely to have a have a positive TST (P<0.001). However, the number of detectable baseline LPA responses to the same mycobacterial preparations was not associated with protection from HIV-associated TB in univariate or multivariate analyses (data not shown).

## Discussion

Among HIV-infected adults in Tanzania we found that the prospective risk of tuberculosis was related to the number and diversity of baseline IFN-γ responses to mycobacterial antigens. All subjects received BCG at birth and most already had latent infection with *M. tuberculosis*. The dominant IFN-γ response was to polyantigenic WCL, and IFN-γ responses to secreted antigens alone were uncommon. These immunologic data fit previously defined conditions associated with immune protection from TB in humans, each of which involves immune challenge with mutiple mycobacterial antigens: TB infection itself [16], [21], infection with non-tuberculous mycobacteria [18], [19], and immunization with polyantigenic mycobacterial vaccines [5], [6], [20], [22].

We hypothesize that the protection from HIV-associated TB observed among subjects with polyantigenic IFN-γ responses to mycobacteria is due to immune responses targeting diverse cell wall, cytosolic and secreted antigens expressed by *M. tuberculosis* during the course of primary, latent and reactivation infection.

Diverse mycobacterial exposures likely contributed to the baseline IFN-γ responses detected in this study. Responses to ESAT-6 signify prior exposure to *M. tuberculosis*, whereas IFN-γ responses to both the heterogeneous mycobacterial antigens in WCL and to Ag85 could arise from prior exposure to *M. tuberculosis,* non-tuberculous mycobacteria (NTM), or BCG immunization [30], [31], [32], [33], [34]. Multiple baseline IFN-γ responses may represent prior exposure to a greater diversity of mycobacterial antigens, including heterologous antigens from mycobacteria other than *M. tuberculosis*. Alternatively, subjects may vary in their IFN-γ responses on the basis of chance preservation of T cell clones reactive to mycobacteria despite progressive HIV infection. Our data do not allow us to differentiate between these possibilities. In the context of vaccine development, we propose that the critical hypothesis to test is whether the most robust marker of protective immunity following immunologic challenge is the number and diversity of both homologous and heterologous mycobacterial antigens eliciting IFN-γ responses.

The risk of incident HIV-associated TB was, as expected, increased in subjects with positive TST [35], but the risk of incident HIV-associated TB was decreased in those with a greater number of baseline IFN-γ responses to mycobacterial preparations. This was true even though both reflect mycobacterial exposure, and TST status and the number of IFN-γ responses against mycobacterial preparations were statistically correlated. We propose two possible explanations for this apparent paradox. First, the TST may be a more specific indication of latent TB than the IFN-γ responses we studied. Alternatively, IFN-γ responses to mycobacterial antigens may be more relevant to host defense against TB than the delayed hypersensitivity reaction represented by a positive TST. In either event, our analyses exclude the possibility that the association between detectable baseline IFN-γ responses against multiple mycobacterial antigens and protection from HIV-associated TB was confounded by TST status since the association remained significant in both the TST positive and TST negative subject subsets.

Baseline IFN-γ responses to the different antigen preparations may not be equivalent or independent in their contribution to protection against subsequent HIV-associated TB. WCL is prepared by disruption of the entire *M. tuberculosis* organism and represents a particularly complex reagent containing innumerable antigens from diverse cellular fractions [28]. Consistent with this, IFN-γ responses to this polyantigenic material were considerably more common than to the other stimuli used and were strongly associated with protection. These findings are concordant with those of a recent study in Pakistan in which IFN-γ responses to *M. tuberculosis* sonicate were more common among subjects with contained latent TB and less common as disease worsened from active pulmonary TB to disseminated TB [36]. The protection from TB associated with IFN-γ responses to these complex mixtures likely reflect immunity targeting multiple protein, lipid or carbohydrate moieties, although our data do not allow us to determine from which cell type(s) these complex responses arise [37], [38]. Our data further suggest that among subjects with IFN-γ responses to WCL concomitant responses to ESAT-6, but not Ag85, augmented the protection from TB. Thus, we hypothesize that interference with ESAT-6-mediated TB pathogenesis is of particular benefit to the host [39].

Strengths of this study include its prospective longitudinal follow up and the use of rigorous published TB criteria for TB diagnosis [13]. A limitation of the study is the possibility that the responses we detected were due solely to the degree of immunosuppression of the HIV-infected subjects in this study. Although we cannot exclude this possibility, the multivariate analysis controlling for CD4 count supports our conclusion that the observed responses were not merely a reflection of underlying HIV-related immunosuppression.

The fact that IFN-γ but not LPA responses to the same antigens were associated with protection suggests a specific mechanistic for IFN-γ responses in immune protection from TB. Lymphocyte proliferation is a generic measure of antigenic sensitization. These responding lymphocytes may have different capacities for producing one or more specific cytokines, including IFN-γ, which was assayed in this study and previously associated with protection from TB in humans [28], [40]. Our findings are consistent with the hypothesis that specific IFN-γ responses to mycobacterial antigens are more closely associated with the underlying immunologic mechanism of protection against TB in humans than more generic tuberculin skin testing or lymphocyte proliferation assays.

Our findings not only contribute to understanding of natural and BCG-induced immunity to TB, but also to the development of new primary and booster vaccines against TB. There are several subunit and whole cell TB vaccine candidates in preclinical and clinical trials development today [41], [42]. Polyantigenic vaccine candidates (e.g., whole cell live or inactivated vaccines) may be more likely than pauci-antigenic candidates (e.g., individual secreted antigens) to induce broad and effective immunity to TB both among HIV-infected subjects with deletion of antigen-specific T cell clones [43], [44], [45] and in healthy subjects with diverse genetic determinants of antigen recognition [46]. In support of this hypothesis, animal data suggest that vaccine induction of IFN-γ responses targeting multiple mycobacterial antigens confers greater protection from TB disease than vaccine induction of IFN-γ responses against single mycobacterial antigens [14], [47], [48]. Further, it has been shown that immunity to TB induced by immunization with inactivated mycobacteria can be adoptively transferred to T cell deficient mice, and that protective antigens are present in cell wall, cytosolic and secreted components of M. tuberculosis [49].

It will be important to determine if subjects immunized with polyantigenic vaccines, including those containing heterologous and potentially cross-protective mycobacterial antigens, exhibit greater vaccine-mediated protection from HIV-associated TB than those immunized with a smaller number of antigens or against exclusively homologous antigens from within the *M. tuberculosis* complex.
